# Elucidating the molecular mechanisms of paeoniflorin intervention in oral lichen planus: a computational biology and bioinformatics–based research strategy

**DOI:** 10.3389/fbinf.2026.1817487

**Published:** 2026-07-17

**Authors:** Tian Zhao, Qi Chen, Liang Yu

**Affiliations:** 1 Stomatological Hospital, General Hospital of Ningxia Medical University, Yinchuan, Ningxia, China; 2 College of Stomatology, Ningxia Medical University, Yinchuan, Ningxia, China; 3 Ningxia Province Key Laboratory of Oral Diseases Research, Yinchuan, Ningxia, China; 4 General Hospital of Ningxia Medical University, Yinchuan, Ningxia, China

**Keywords:** bioinformatics, computational biology, molecular dynamics simulation, oral lichen planus, paeoniflorin

## Abstract

**Background:**

Oral lichen planus (OLP) is a chronic inflammatory mucosal disease with a risk of malignant transformation and limited long-term therapeutic options. Paeoniflorin (PF), a natural monoterpene glycoside, exhibits multi-target anti-inflammatory and immunomodulatory properties, but its systematic mechanisms against OLP remain elusive.

**Methods:**

We employed an integrative framework combining network pharmacology, transcriptomic cross-validation, molecular docking, and molecular dynamics (MD) simulations. Public databases were mined to identify PF targets and OLP-related genes. Core targets were prioritized via protein-protein interaction (PPI) network topology and further validated using OLP tissue transcriptomic datasets (GSE52130 and GSE213349). Functional enrichment analyses were performed, followed by structural validation of PF–target binding via molecular docking and 100-ns MD simulations.

**Results:**

Sixty-eight overlapping targets between PF and OLP were identified. PPI network analysis and transcriptomic cross-validation pinpointed eight core targets: AKT1, IL6, MMP9, STAT3, TNF, IL1B, PTGS2, and PDE4B. Mechanistically, these targets converged on the TNF, PI3K–Akt, and MAPK signaling pathways, regulating inflammatory response, cell migration, apoptosis, and protease activity at membrane microdomain and extracellular matrix interfaces. Molecular docking showed PF binding affinities comparable to or exceeding reference inhibitors (e.g., STAT3: −9.27 vs. Stattic −9.16 kcal/mol). MD simulations confirmed stable conformational binding, with the STAT3 and PDE4B complexes exhibiting the most balanced rigidity and lowest ligand RMSD (0.09–0.10 nm).

**Conclusion:**

This study provides a systems-level map of PF’s multi-target intervention in OLP, highlighting a composite anti-inflammatory–immune reprogramming–pro-repair axis centered on core inflammatory kinases and proteases. The structural validation of key targets establishes a mechanistic rationale for PF as a promising therapeutic candidate, warranting further preclinical and clinical development for OLP management.

## 1 Introduction

Oral lichen planus (OLP) is a mucosal disorder characterized by chronic mucosal inflammation and immune-mediated epithelial injury, clinically presenting with polymorphic lesions such as reticular, plaque-like, and erosive/ulcerative patterns, with a relapsing–remitting course that predominantly affects middle-aged women (Louisy et al., 2024). Beyond its substantial impact on quality of life, OLP carries a non-negligible risk of malignant transformation, underscoring the unmet need for safer, more effective, and sustainably maintainable therapeutic strategies. Current first-line therapy relies on topical corticosteroids (Lodi et al., 2020), supplemented by calcineurin inhibitors, retinoids, and laser or phototherapy modalities (Hesse et al., 2020; Mutafchieva et al., 2018). However, long-term management is limited by high recurrence rates and adverse effects such as mucosal atrophy and secondary infections, and clinical responses remain inconsistent in recalcitrant erosive phenotypes or in patients with prominent pain and burning sensations. Achieving durable inflammatory control while re-establishing mucosal homeostasis—without accumulating treatment-related harm—remains a central therapeutic challenge in OLP (Krupaa et al., 2015; Spirito et al., 2024; Selaru et al., 2023).

Mechanistically, OLP exhibits a systems-level, multi-node coupling of inflammatory–immune dysregulation (El-Howati et al., 2023). T cell–mediated basal keratinocyte apoptosis is amplified by Th1/Th17 predominance and insufficient Treg function; proinflammatory cytokines, including TNF-α, IFN-γ, IL-6, and IL-17, are persistently upregulated, accompanied by activation of NF-κB, MAPK, and JAK/STAT signaling pathways (Lu et al., 2015; Weaver et al., 2013; Xu et al., 2021; Marabi et al., 2024). Impaired mucosal barrier repair, aberrant angiogenesis, and neurogenic inflammation further perpetuate chronicity. This multidimensional pathological network suggests that single-target interventions are unlikely to yield robust and durable efficacy, whereas an integrated strategy—encompassing anti-inflammatory activity, immune reprogramming, antioxidation, and pro-repair actions—may be both theoretically sound and practically feasible (Kurago, 2016; White et al., 2019).

Against this backdrop, natural products have attracted interest due to their multi-target pharmacology, low toxicity, and suitability for long-term administration. Paeoniflorin, a representative bioactive constituent of Paeonia species, has accumulated translational evidence in OLP-related research, indicating a promising direction for further development. Pharmacological and mechanistic studies suggest that paeoniflorin can suppress innate immune responses by reducing IL-1β, IL-6, and TNF-α release and attenuating intracellular inflammation via NF-κB inhibition; at the adaptive immunity level, it promotes rebalancing of the Th1/Th17–Treg axis; with respect to oxidative stress, it activates the Nrf2/HO-1 pathway, scavenges reactive oxygen species, and preserves mitochondrial function (Zhang Z. et al., 2022). In parallel, paeoniflorin has been shown to reduce mucosal microvascular permeability and to inhibit MMP-9– and VEGF-mediated positive feedback linking inflammation to remodeling, while bidirectionally modulating keratinocyte apoptosis and autophagy (Zhu et al., 2017; Feng et al., 2025; Takeuchi et al., 2020). These routes of action align closely with key nodes along the OLP pathogenic chain, conferring a “pathology-aligned” theoretical advantage (Kurt et al., 2019; Ni et al., 2016). At the preclinical level, oral keratinocytes exposed to OLP-mimicking inflammatory stimuli (TNF-α, IFN-γ, or LPS) exhibit upregulation of proinflammatory mediators, increased adhesion molecule expression, and heightened oxidative stress; paeoniflorin intervention significantly reduces IL-6, IL-8, and COX-2 expression, inhibits IκBα phosphorylation and p65 nuclear translocation, and, in some studies, suppresses STAT3 activation and NLRP3 assembly—demonstrating multi-tier blockade along the “inflammatory sensing–signal transduction–effector” axis (Zhao et al., 2018; Cao and Yang, 2022; Yu et al., 2019; Jia and He, 2016; Cai et al., 2024). Collectively, these observations resonate with OLP pathology and show a degree of consistency and reproducibility, strengthening the mechanistic plausibility and translational potential of paeoniflorin in this indication (Zhang and Wei, 2020).

Nevertheless, the multi-target and multi-pathway nature of paeoniflorin also complicates mechanistic dissection. Which targets occupy “hub” positions within the OLP-specific molecular landscape? Do key pathways differ across clinical phenotypes (e.g., erosive versus reticular) and immune infiltration patterns? How do cross-pathway synergy or antagonism shape overall efficacy and safety? Moreover, in the absence of robust animal models, how can these mechanistic hypotheses be validated and prioritized within the authentic molecular context of human lesions to achieve a closed loop of interpretability? These questions represent critical bottlenecks for high-quality translation of paeoniflorin.

To address these challenges, systems-level strategies grounded in computational biology and bioinformatics offer distinctive advantages (Zhang Y. et al., 2022; Batzoglou and Schwartz, 2014). By integrating public transcriptomic datasets, disease-related gene and pathway repositories, protein interaction networks, immune cell infiltration estimates, and functional enrichment analyses, one can establish quantitative and interpretable links across four tiers: the disease molecular atlas, putative drug targets, pathway redundancy and coupling, and prioritization of critical nodes. In combination with network pharmacology, molecular docking, and molecular dynamics simulations, drug–target compatibility can be verified from structural and functional perspectives. Further, approaches such as weighted gene co-expression network analysis can identify modules associated with clinical phenotypes and cross-validate them against the paeoniflorin action network, thereby pinpointing core axes characterized by high contribution, druggability, and safety. This integrated framework not only elucidates the molecular mechanisms of paeoniflorin in OLP but also provides a rational basis for the development of potential companion diagnostic biomarkers, the identification of responsive subpopulations, and the design of rational combination therapies.

Building on the above, we propose an integrated framework—disease molecular feature mapping → drug action network inference → structural-level validation → data-driven back-validation—to systematically interrogate the molecular mechanisms by which paeoniflorin intervenes in OLP. The specific objectives are as follows: (i) to delineate, within the transcriptomic context of *bona fide* human lesions, the core pathways of inflammation–immunity–oxidative stress together with the immune infiltration landscape of OLP; (ii) to integrate bioinformatics and computational biology for the prediction and prioritization of paeoniflorin’s putative therapeutic targets and key pathways, thereby resolving its multi-target–multi-pathway–multi-tier logic of action; (iii) to construct a drug–disease interaction network and identify hub nodes, followed by structural validation *via* molecular docking and molecular dynamics simulations; and (iv) to perform cross-validation constrained by independent datasets and existing experimental evidence, achieving a closed loop of interpretability for mechanistic inference. We anticipate that this study will elucidate, at a systems level, the composite anti-inflammatory–immune reprogramming–antioxidant–pro-repair axis of paeoniflorin in OLP, enhance the scientific rigor and predictability of its translational application, and provide a reproducible research paradigm for precision intervention in chronic inflammatory diseases of the oral mucosa.

## 2 Materials and methods

### 2.1 Network pharmacology analysis

We integrated multi-source databases (Table 1) to systematically delineate the molecular network through which paeoniflorin may act against oral lichen planus (OLP). The workflow comprised the following steps.

**TABLE 1:** Databases and tools employed in network pharmacology analysis.

Abbreviation name	Full name and description	Website address
SEA	Similarity ensemble approach	https://sea.bkslab.org/
UniProt	The universal protein knowledgebase	https://www.uniprot.org
OMIM	Online mendelian inheritance in man	https://www.omim.org/
GeneCards	The human gene database	https://www.genecards.org/
BATMAN	BATMAN-TCM	http://bionet.ncpsb.org.cn/batman-tcm/#/home
DrugBank	A vital resource for pharmaceutical research	https://go.drugbank.com/
Bioinformatics	A free online platform for data visualization and graphing	https://www.bioinformatics.com.cn
TTD	Therapeutic target database	https://db.idrblab.net/ttd/
STRING	STRING protein-protein interaction networks functional enrichment analysis	https://string-db.org/
Metascape	A biologist-oriented resource for the analysis of systems-level datasets	https://metascape.org/gp/index.html#/main/step1
TCMSP	Traditional Chinese medicine systems pharmacology database and analysis platform	http://lspnwu.edu.cn/tcmspphp
TargetNet	An open web server that could be used for netting or predicting the binding of multiple targets for any given molecule	http://targetnet.scbdd.com/
Cytoscape3.9.1	Cytoscape: a Software environment for integrated models of biomolecular interaction networks	https://cytoscape.org/
Swiss target prediction	A tool that predicts the biological targets of small molecules based on their SMILES or structure	http://www.swisstargetprediction.ch/
PharmMapper	An updated integrated pharmacophore matching platform with statistial method for potential target identification	https://www.lilabecust.cn/pharmmapper/
DISGENET	A comprehensive knowledge database integrating and standardizing information on disease associated genes and variants	https://www.disgenet.com

First, compound and disease targets were collected and standardized. Putative targets of paeoniflorin were predicted using the following databases with explicit filtering criteria: PharmMapper (species limited to *Homo sapiens*; normalized fit score ≥0.8), TargetNet (probability ≥0.5), SwissTargetPrediction (probability ≥0.1), BATMAN-TCM (score cutoff >20, P < 0.05), and TCMSP (all targets linked to paeoniflorin were included without additional filtering). Known targets were retrieved from DrugBank. All collected targets were harmonized to official gene symbols according to UniProt (Cui et al., 2025; Ru et al., 2014; Knox et al., 2011; Wei et al., 2026). Oral lichen planus (OLP)-associated targets were retrieved using the keyword “Oral lichen planus” from GeneCards (relevance score ≥20), DisGeNET (score ≥0.2), OMIM, and TTD; the resulting entries were merged and de-duplicated to construct the disease target set (Stelzer et al., 2016; Wang et al., 2026). Overlapping targets between paeoniflorin and OLP were identified by Venn diagram analysis.

The overlapping targets were imported into the STRING database (Szklarczyk et al., 2025) (minimum required interaction score ≥0.90; organism set to *H. sapiens*; nodes with zero degree/disconnected nodes hidden) to construct a protein–protein interaction (PPI) network. To prioritize core targets potentially modulated by the compound in oral lichen planus, network topology was analyzed in Cytoscape 3.9.1 using the CytoNCA plugin, calculating four indices: degree centrality, betweenness centrality, closeness centrality, and network centrality. In parallel, CytoHubba was employed to rank nodes using the Maximal Neighborhood Component (MNC), Maximal Clique Centrality (MCC), and Edge Percolated Component (EPC) algorithms. The top 10 targets under each metric were then intersected via Venn analysis to define the core target set (Doncheva et al., 2023).

Finally, functional annotation of the common targets was conducted using Metascape to elucidate mechanisms underlying the therapeutic action of paeoniflorin in OLP (Zhou et al., 2019). For Gene Ontology (GO) enrichment, analyses were restricted to *H. sapiens*, with a significance cutoff of p < 0.01 and a minimum set size of at least three genes; the statistical significance of the enrichment results was adjusted using the Benjamini–Hochberg method to control the false discovery rate (FDR). Terms with a *q*-value <0.05 were considered significantly enriched. Results were interpreted across three domains: biological process (BP) emphasized target involvement in signaling cascades and regulatory circuitry; molecular function (MF) focused on protein-binding properties and changes in enzymatic activity; and cellular component (CC) delineated the subcellular localization of targets. For Kyoto Encyclopedia of Genes and Genomes (KEGG) pathway enrichment (Kanehisa et al., 2016; Kanehisa and Goto, 2000; Kanehisa et al., 2025; Kanehisa, 2019), the above baseline settings were retained, and an additional filter of enrichment factor >1.5 was applied. *q*-values were transformed by log10, and pathways most relevant to oral lichen planus with higher gene counts were visualized using bubble plots, where bubble size reflects the number of enriched genes and color gradients indicate the level of *q*-value significance.

### 2.2 Molecular docking

Docking was performed with AutoDock Vina, using MGLTools for preprocessing; Open Babel for energy minimization; and PyMOL together with PLIP/LigPlot + for visualization and interaction analysis. All computations were run under Ubuntu (see Supplementary Material for procedural details).

The ligand, paeoniflorin, was retrieved from PubChem, protonated at pH 7.4, assigned Gasteiger charges, and its rotatable bonds were defined prior to export in PDBQT format. Protein receptors were obtained from the Protein Data Bank, with co-crystallized ligands and non-essential water molecules removed; hydrogens and charges were then assigned. Docking grids were defined around the centroid of the co-crystallized ligand and any annotated pocket to ensure comprehensive coverage of the active site (Chang et al., 2019). Interaction profiles (hydrogen bonds, hydrophobic contacts, etc*.*) were parsed in PyMOL, and random seeds and full logs were recorded to ensure reproducibility (Rosignoli et al., 2023).

### 2.3 Molecular dynamics simulations

Molecular dynamics (MD) simulations were conducted in Schrödinger Desmond using the OPLS4 force field (Lu et al., 2021). The top-ranked protein–ligand complexes from docking served as starting structures. Proteins were prepared with the Protein Preparation Workflow, and protonation states were assigned at pH 7.4 using Epik/PROPKA; the ligand was processed with LigPrep at pH 7.4. Systems were constructed with the System Builder in an explicit TIP3P water box (10 Å buffer to the solute boundary), neutralized and set to 0.15 M NaCl, with long-range electrostatics treated by particle mesh Ewald (PME) (Rosas Jimenez et al., 2024). After relaxation via the Relax Model System protocol (energy minimization and short pre-equilibration), production runs were carried out in the NPT ensemble (310 K, 1 bar) using a Nosé–Hoover thermostat and a Martyna–Tobias–Klein barostat. The integration time step was 2 fs (extended to 4 fs when hydrogen mass repartitioning was applied); bonds involving hydrogens were constrained with M-SHAKE. Each system was simulated for 100 ns; coordinates were saved every 10 ps and energies every 2 ps. Simulation quality and convergence were monitored using Simulation Interaction Diagram panels, tracking temperature, pressure, density, potential energy, and Cα/ligand RMSD. Convergence was defined by RMSD.

## 3 Results and discussion

### 3.1 Network pharmacology analysis

#### 3.1.1 Collection and screening of compound and disease targets

**FIGURE 1:**
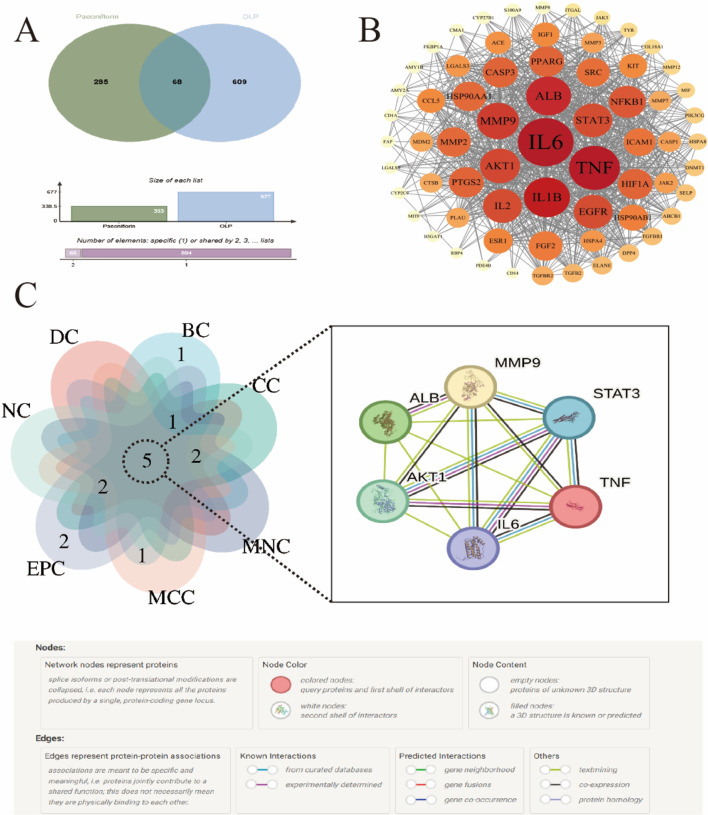
Network pharmacological analysis of paeoniflorin and oral lichen planus. **(A)** Venn diagram of drug and disease targets, The overlapping area represents 68 common potential targets; **(B)** Protein-protein interaction (PPI) network of common drug-disease targets, Nodes represent proteins, and edges represent the interactions between them; **(C)** Flowchart of core target screening The workflow illustrates the criteria and process for identifying the core targets from the PPI network.

Database mining identified 353 putative targets for the compound. Intersection with oral lichen planus (OLP)-related targets yielded 68 overlapping targets (Figure 1A), representing the potential nodes through which the compound may modulate OLP. A protein–protein interaction (PPI) network was constructed based on these overlapping targets (Figure 1B), comprising 66 nodes and 885 edges. In the visualization, nodes represent proteins and edges denote protein–protein interactions; node size and color intensity are positively scaled to degree, such that larger and darker nodes indicate higher connectivity.

#### 3.1.2 PPI network topological analysis and identification of core targets

Topological analysis of the PPI network using CytoNCA and CytoHubba initially identified several highly connected nodes. Among these, albumin (ALB) ranked prominently in certain centrality metrics. However, given that ALB is well established as a carrier protein and is not generally considered a direct pharmacological target, it was excluded from the core therapeutic target list. Accordingly, the core network hubs retained for downstream functional analysis were AKT1, IL6, MMP9, STAT3, and TNF (Figure 1C; Table 2). These targets are closely implicated in OLP pathophysiology: AKT1 participates in autophagy and metabolic regulation; IL6 and TNF mediate inflammatory responses; MMP9 is involved in immune and extracellular matrix remodeling.

**TABLE 2:** Network topology parameters of the core targets.

*NO.*	Gene name	Network topological parameter						
		Betweenness	Closeness	MNC	Network	Degree	MCC	EPC
1	AKT1	93.85	0.78	47	43.17	47	3.83 × 10^20^	16.19
2	IL6	357.39	0.91	59	56.88	59	3.83 × 10^20^	16.83
3	MMP9	114.65	0.80	49	45.72	49	3.83 × 10^20^	15.80
4	STAT3	80.71	0.77	46	41.94	46	3.83 × 10^20^	16.06
5	TNF	281.87	0.90	58	56.06	58	3.83 × 10^20^	16.90
6	ALB	183.81	0.83	52	48.92	52	3.83 × 10^20^	15.44

#### 3.1.3 Transcriptomic cross-validation against oral mucosa data

To establish spatial and disease-specific biological relevance, the 68 overlapping targets were cross-referenced with publicly available oral mucosa transcriptomic data. Differentially expressed genes (DEGs) were identified from the integrated GSE52130 and GSE213349 datasets using the thresholds |log_2_ fold change| > 1 and adjusted P < 0.05, yielding 616 DEGs. Venn diagram (Supplementary Figure S1, and Supplementary Figure S2) analysis between these 616 DEGs and the 68 network pharmacology-predicted targets revealed three intersecting genes: IL1B, PTGS2, and PDE4B. These three targets, all of which exhibited significant dysregulation in oral lichen planus tissues, were therefore adopted as biologically corroborated disease-relevant targets. Collectively, the final set of core targets subjected to subsequent molecular docking and molecular dynamics simulations comprised (i) five hub targets derived from PPI network topology (TNF, STAT3, IL6, MMP9, AKT1) and (ii) three transcriptionally validated targets (IL1B, PTGS2, PDE4B). This integrative strategy couples network-level centrality with tissue-level expression evidence, thereby strengthening the biological plausibility of the selected targets for paeoniflorin against oral lichen planus.

#### 3.1.4 GO enrichment analysis

**FIGURE 2:**
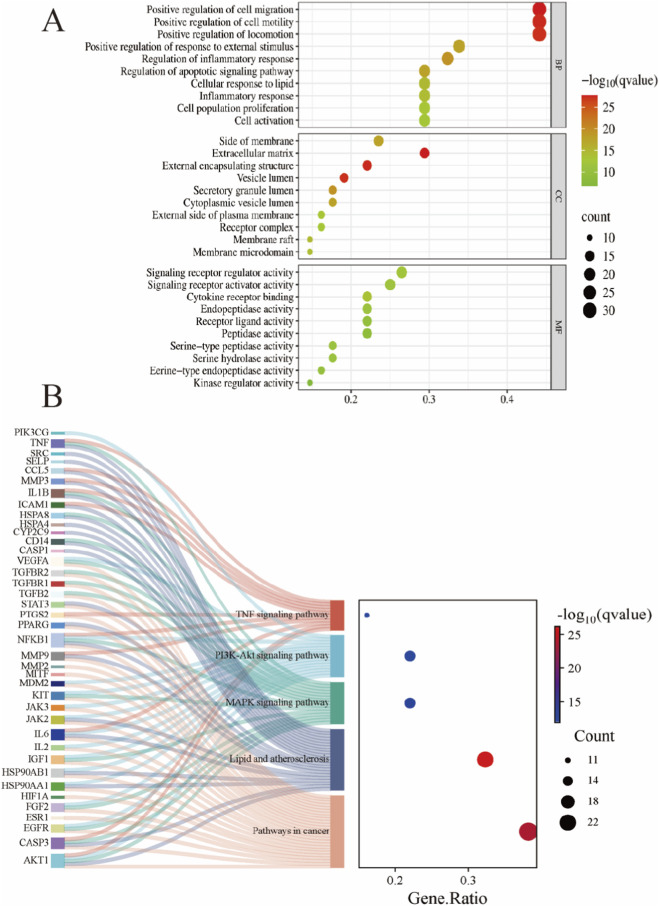
GO and KEGG analysis. **(A)** Gene Ontology (GO) enrichment analysis of common targets The bar plot shows the significantly enriched terms in biological processes (BP), molecular functions (MF), and cellular components (CC); **(B)** KEGG pathway enrichment analysis of common targets. Sankey diagram shows pathway-gene correspondence (link width indicates gene count); bubble plot displays enrichment significance (bubble size represents gene number, color indicates -log10 (*q*-value).

GO enrichment indicates that the putative intervention network of paeoniflorin in oral lichen planus (OLP) converges on key biological themes encompassing cell migration/activation, regulation of inflammation and apoptosis, and lipid-associated responses, with spatial coordination across membrane receptor–microdomain platforms, the secretory vesicle–matrix interface, and the extracellular microenvironment (Figure 2A). Specifically, within the biological process (BP) category, enrichment in positive regulation of cell migration/motility/locomotion alongside cell population proliferation and cell activation highlights regulatory nodes governing the dynamic behaviors of immune cells and epithelial cells—features consistent with OLP lesions characterized by T-cell infiltration, keratinocyte stress, and microenvironmental remodeling. Concurrent enrichment in regulation of inflammatory response, inflammatory response, and positive regulation of response to external stimulus suggests that paeoniflorin may recalibrate the input and threshold of proinflammatory signaling and stress responsiveness to restore homeostasis in chronically inflamed mucosa. The presence of regulation of apoptotic signaling pathway implies a capacity to restrain pathological apoptosis or re-establish the coupling between survival and death signals, a mechanism pertinent to mitigating basal cell degeneration and barrier disruption. Notably, cellular response to lipid integrates lipid metabolism with the immune–inflammatory axis, implying potential effects on lipid-mediated receptor signaling, membrane biophysical properties, and redox–inflammation crosstalk.

At the cellular component (CC) level, targets are significantly enriched in the extracellular matrix, external encapsulating structure, and external side of plasma membrane, linking the intervention network to matrix remodeling, transmembrane receptor transduction, and events at the cell–extracellular interface. Overrepresentation in membrane raft/microdomain and receptor complex points to receptor clustering and signal integration on lipid platforms as critical control points, in line with the microdomain dependence of TNF, growth factor, and chemokine signaling. Enrichment in vesicle lumen, secretory granule lumen, and cytoplasmic vesicle lumen underscores the importance of secretory and degranulation routes (e.g., cytokine and protease release) in disease progression and intervention, suggesting that paeoniflorin may attenuate inflammatory amplification by modulating vesicular trafficking and exocytosis.

In the molecular function (MF) category, enrichment in cytokine receptor binding, signaling receptor regulator activity, signaling receptor activator activity, and receptor ligand activity delineates multi-layered interfaces at the ligand–receptor–regulator level, indicating modulation not only of downstream kinases but also of receptor sensitivity and threshold setting upstream. Concurrent enrichment of endopeptidase activity, serine-type peptidase activity, serine hydrolase activity, and peptidase activity connects the network to proteolytic programs involved in extracellular matrix degradation, antigen processing, and maturation of inflammatory mediators, suggesting that paeoniflorin may constrain serine protease/endopeptidase activity to reduce tissue damage and mediator activation. Finally, enrichment of kinase regulator activity focuses attention on regulatory control over key axes such as PI3K/AKT and MAPK, thereby establishing coverage across the receptor–kinase–effector continuum.

#### 3.1.5 KEGG pathway enrichment analysis

KEGG enrichment ranked the TNF signaling pathway, PI3K–Akt signaling pathway, MAPK signaling pathway, Lipid and atherosclerosis, and Pathways in cancer among the top five terms (Figure 2B), delineating a signaling topology highly concordant with the GO results. The TNF pathway is a principal driver of chronic mucosal inflammation and tissue injury in OLP; clustering of upstream receptors within membrane microdomains initiates NF-κB and MAPK cascades, amplifies cytokine networks, and engages apoptosis/necroptosis-associated programs. The concurrent enrichment of targets in cytokine receptor binding, receptor regulator activity, and the cellular components membrane raft/receptor complex provides orthogonal corroboration, suggesting that paeoniflorin may attenuate inflammatory amplification by diminishing receptor signalosome activity or remodeling microdomain architecture. The PI3K–Akt pathway, tightly linked to cell survival, motility, metabolism, and anti-apoptotic signaling, aligns directly with BP terms such as regulation of apoptotic signaling pathway and positive regulation of cell migration/motility, indicating that paeoniflorin may re-establish a dynamic equilibrium between keratinocyte survival signaling and the containment of pathological migration/hyperproliferation. As a core stress–inflammation conduit, the MAPK pathway integrates responses to external stimuli with inflammatory regulation and protease expression; together with MF enrichment for kinase regulator activity and endopeptidase/serine-type peptidase activity, this pattern supports the hypothesis that dampening ERK/p38/JNK activity and its downstream protease network could mitigate extracellular matrix degradation and maladaptive tissue remodeling.

Enrichment of Lipid and atherosclerosis couples lipid signaling, oxidative stress, and inflammation, consistent with BP terms (cellular response to lipid) and CC annotations (membrane raft/microdomain), and points to modulation of lipid-mediated receptor signaling and the membrane milieu as a means to reduce inflammatory sensitivity and adhesion–migration phenotypes in the mucosal microenvironment. Finally, enrichment in Pathways in cancer should be interpreted as convergence on core modules governing proliferation, apoptosis evasion, and epithelial plasticity rather than an oncogenic action *per se*. In the clinical context of OLP, where malignant transformation risk exists, this suggests that paeoniflorin could broadly tune PI3K–Akt/MAPK axes to suppress pro-proliferative and pro-migratory programs.

Integrating the KEGG and GO evidence yields a systems-level mechanistic model for paeoniflorin in OLP. Spatially, the intervention is centered on receptor complexes within membrane microdomains and at the extracellular matrix–secretory vesicle interface. Functionally, it operates across three interconnected tiers: (i) regulation of cytokine receptor signaling at the membrane, (ii) modulation of kinase axes (PI3K–Akt/MAPK), and (iii) restraint of proteolysis and matrix remodeling, with a parallel branch coupling lipid responses to inflammatory amplification. Within this framework, paeoniflorin is expected to downshift TNF and related proinflammatory circuits, reset receptor sensitivity and kinase activation thresholds, inhibit serine protease–driven tissue damage, and rebalance migration/survival versus apoptotic signaling, thereby correcting the chronic inflammatory and remodeling disequilibrium in OLP through multi-target, network-level modulation. These insights propose clear avenues for experimental validation, including assays of receptor clustering and raft stability, AKT/ERK phosphorylation dynamics, protease activity and matrix degradation markers, and lipid-derived inflammatory metabolite profiling.

### 3.2 Analysis of molecular docking results

**FIGURE 3:**
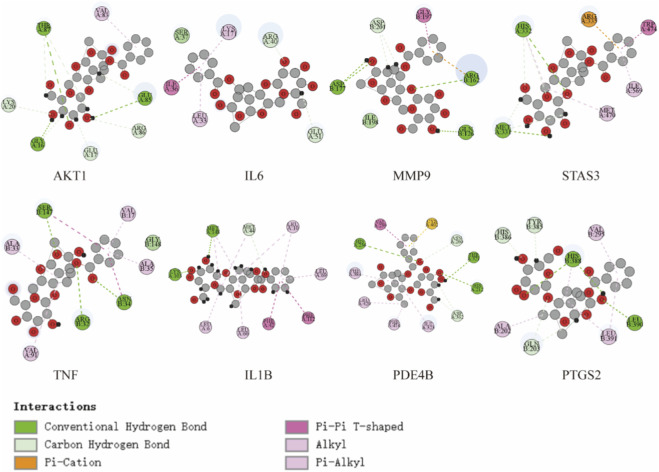
Molecular docking results of paeoniflorin with core disease targets. Distinct colored blocks denote different types of interactions between the compound and functional residues.

The AutoDock Vina binding free energies of paeoniflorin against the eight core targets, together with those of the corresponding reference inhibitors, are summarized in Table 3 and Figure 3. Across all targets, paeoniflorin exhibited favorable binding affinities ranging from −6.29 to −9.80 kcal/mol. Notably, its predicted affinities for AKT1 (−7.91 kcal/mol), IL6 (−7.30 kcal/mol), STAT3 (−9.27 kcal/mol), IL1B (−8.96 kcal/mol), and PDE4B (−7.22 kcal/mol) were comparable to or exceeded those of the respective reference inhibitors (Ipatasertib −7.31, LMT-28–6.58, Stattic −9.16, MCC950–6.04, and Rolipram −6.19 kcal/mol). For MMP9, TNF, and PTGS2, paeoniflorin showed reasonably strong binding (−6.29, −9.80, and −7.91 kcal/mol, respectively), though slightly weaker than Batimastat (−8.38), SPD304 (−10.43), and Celecoxib (−10.26 kcal/mol). Detailed interaction fingerprints for each complex are provided in the Supplementary Material. Overall, paeoniflorin consistently employs a convergent recognition motif combining conventional/carbon hydrogen bonds for anchoring, π-stacking for site selectivity, and hydrophobic contacts for steric complementarity. This multi-target engagement profile, validated by quantitative comparison with known inhibitors, supports the structural plausibility of paeoniflorin as a systems-level modulator in oral lichen planus.

**TABLE 3:** Molecular docking binding affinities of paeoniflorin and reference inhibitors against selected targets.

Target	PDB ID	Paeoniflorin affinity	Reference inhibitor	Ref. Affinity
AKT1	1UNQ	−7.91	Ipatasertib	−7.31
IL6	1IL6	−7.30	LMT-28	−6.58
MMP9	1GKC	−6.29	Batimastat	−8.38
STAT3	6NJS	−9.27	Stattic	−9.16
TNF	1TNF	−9.80	SPD304	−10.43
IL1B	1I1B	−8.96	MCC950	−6.04
PDE4B	1ZKL	−7.22	Rolipram	−6.19
PTGS2	5F19	−7.91	Celecoxib	−10.26

All affinity values are expressed in kcal/mol.

### 3.3 Molecular dynamics simulation analysis

**FIGURE 4:**
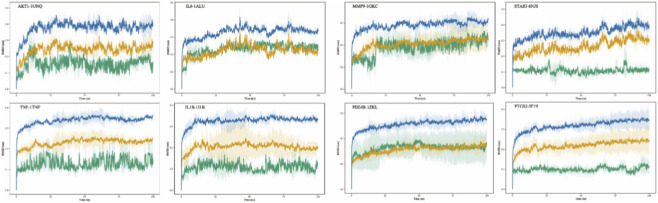
RMSD profiles for paeoniflorin–core target protein complexes. An RMSD fluctuation within 0.1–1 nm sustained for more than 50 ns is generally considered indicative of a stable receptor–ligand association.

**FIGURE 5:**
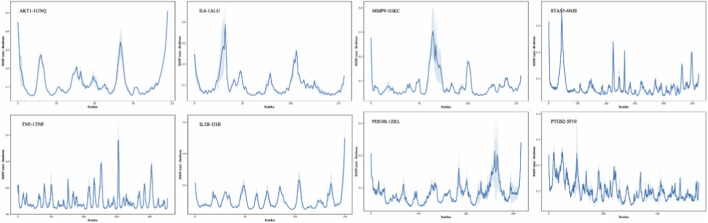
RMSF profiles for paeoniflorin–core target protein complexes.

Based on three independent molecular dynamics simulations per complex, all paeoniflorin–target protein complexes achieved satisfactory equilibration over the 100 ns time scale (Figure 4; Supplementary Table S1). The RMSD of each complex entered a stable plateau after a brief initial rise: the AKT1, IL6, and MMP9 complexes converged within the first 25 ns, 12 ns, and 23 ns, respectively, whereas the remaining systems (STAT3, TNF, IL1B, PDE4B, and PTGS2) displayed low standard deviations of RMSD, indicating stable conformational fluctuations throughout the simulations. Comparison of the average RMSD values revealed that the STAT3 complex exhibited the highest overall rigidity (0.19 nm), followed by PDE4B (0.25 nm) and MMP9 (0.29 nm). The AKT1 complex showed the largest average RMSD of 0.37 nm, while the IL6, TNF, IL1B, and PTGS2 complexes fell within the range of 0.31–0.33 nm, all remaining within an acceptable stability range. The ligand paeoniflorin remained highly stable in most systems; in particular, when bound to STAT3, IL1B, and PTGS2, its average RMSD was only 0.09–0.10 nm, with almost no conformational drift. In the IL6 system, paeoniflorin showed a slightly higher RMSD of 0.23 nm, suggesting that the binding mode may have undergone a certain degree of local adjustment. Regarding the receptor proteins, PDE4B exhibited the lowest RMSD (0.15 nm). Although the average RMSD of IL1B was 0.20 nm, its standard deviation reached 0.051 nm, reflecting substantial conformational sampling heterogeneity of this protein during the simulation. RMSF (Figure 5) analysis further disclosed differences in local flexibility: residue fluctuations in the IL1B, PDE4B, and PTGS2 systems were generally stable, with the maximum RMSF not exceeding 0.2 nm, indicating that the protein backbones maintained high rigidity upon paeoniflorin binding. In contrast, residues 48–52 of STAT3 and residues 302–306 of TNF reached RMSF values as high as 0.6 nm, while residues 25–30 of IL6 and residues 82–87 of AKT1 reached 0.4 nm and 0.3 nm, respectively. These highly flexible regions generally corresponded to surface loops, and their large-amplitude motions did not completely disrupt the overall stability of the complexes. Notably, in the STAT3 system, despite exhibiting the highest local RMSF, both the complex and the receptor protein retained low average RMSD values of 0.19 nm, indicating that the flexible loop region did not compromise the tight packing of the binding interface. Collectively, the complexes formed by paeoniflorin with STAT3 and PDE4B featured both low overall RMSD and low or localized residue fluctuations, displaying the most balanced conformational stability. Although the AKT1 and IL6 systems were overall stable, their relatively large RMSD values and pronounced local flexibility suggest that their binding modes may involve dynamically induced conformational selection.

## 4 Conclusion

This study provides a systems-level mechanistic framework for the action of paeoniflorin (PF) in oral lichen planus (OLP) by integrating transcriptomic profiling, network pharmacology, and structural simulations. Our analysis identified a core target hub—comprising TNF, STAT3, IL6, MMP9, AKT1, IL1B, PTGS2, and PDE4B—that converges on the TNF, PI3K–Akt, and MAPK signaling pathways at membrane raft and extracellular matrix interfaces. Molecular docking and 100-ns molecular dynamics simulations further demonstrated that PF forms conformationally stable complexes with these targets, exhibiting binding affinities comparable to known inhibitors, with the STAT3 and PDE4B complexes showing particularly robust structural stability. These findings provide a network-level and structural rationale that supports the further investigation of PF as a multi-target candidate for OLP. However, it must be emphasized that the target prediction and pathway enrichment analyses presented here are entirely computational in nature, derived from publicly available databases without independent experimental validation using OLP patient samples or PF-treated cellular models. The accuracy of the identified targets is therefore constrained by the completeness of the underlying databases and the predefined screening thresholds. Given this lack of direct experimental confirmation and the absence of clinical data or biomarker-based stratification, the current conclusions should be regarded as hypothesis-generating rather than as evidence for a precision intervention. Future work integrating independent molecular validation, phenotype-stratified transcriptomics, and functional assays will be essential to substantiate and refine these mechanistic insights.

## Data Availability

The original contributions presented in the study are included in the article/Supplementary Material, further inquiries can be directed to the corresponding author.

## References

[B1] Batzoglou S. Schwartz R. (2014). Computational biology and bioinformatics. Bioinformatics 30 (12), i1–i2. 10.1093/bioinformatics/btu304 24931972 PMC4058912

[B2] Cai C. Liu S. Liu Y. Huang S. Lu S. Liu F. (2024). Paeoniflorin mitigates insulin-like growth factor 1-induced lipogenesis and inflammation in human sebocytes by inhibiting the PI3K/Akt/FoxO1 and JAK2/STAT3 signaling pathways. Nat. Prod. Bioprospect 14 (1), 56. 10.1007/s13659-024-00478-4 39349732 PMC11442718

[B3] Cao L. Yang K. (2022). Paeoniflorin attenuated TREM-1-Mediated inflammation in THP-1 cells. J. Healthc. Eng. 2022, 7051643. 10.1155/2022/7051643 35480155 PMC9038380

[B4] Chang D. Sun S. Zhang C. (2019). An accelerated linearly convergent stochastic L-BFGS algorithm. IEEE Trans. Neural Netw. Learn Syst. 30 (11), 3338–3346. 10.1109/TNNLS.2019.2891088 30703047

[B5] Cui Y. Peng C. Xia Z. Yang C. Guo Y. (2025). A survey of sequence-to-graph mapping algorithms in the pangenome era. Genome Biol. 26 (1), 138. 10.1186/s13059-025-03606-6 40405275 PMC12096488

[B6] Doncheva N. T. Morris J. H. Holze H. Kirsch R. Nastou K. C. Cuesta-Astroz Y. (2023). Cytoscape stringApp 2.0: analysis and visualization of heterogeneous biological networks. J. Proteome Res. 22 (2), 637–646. 10.1021/acs.jproteome.2c00651 36512705 PMC9904289

[B7] El-Howati A. Thornhill M. H. Colley H. E. Murdoch C. (2023). Immune mechanisms in oral Lichen planus. Oral Dis. 29 (4), 1400–1415. 10.1111/odi.14142 35092132

[B9] Feng R. Tian F. Zhou J. Ping Y. Han W. Shi X. (2025). A preliminary study on the promotion of wound healing by paeoniflorin carbon dots loaded in chitosan hydrogel. Biomed. Mater 20 (3), 1748. 10.1088/1748-605x/add2ba 40306299

[B10] Hesse J. Schmalfuss A. Kvaal S. I. (2020). Photodynamic therapy of oral Lichen planus. Photochem Photobiol. Sci. 19 (10), 1271–1279. 10.1039/d0pp00249f 32945823

[B11] Jia Z. He J. (2016). Paeoniflorin ameliorates rheumatoid arthritis in rat models through oxidative stress, inflammation and cyclooxygenase 2. Exp. Ther. Med. 11 (2), 655–659. 10.3892/etm.2015.2908 26893662 PMC4734021

[B13] Kanehisa M. (2019). Toward understanding the origin and evolution of cellular organisms. Protein Sci. 28 (11), 1947–1951. 10.1002/pro.3715 31441146 PMC6798127

[B14] Kanehisa M. Goto S. (2000). KEGG: kyoto encyclopedia of genes and genomes. Nucleic Acids Res. 28 (1), 27–30. 10.1093/nar/28.1.27 10592173 PMC102409

[B15] Kanehisa M. Sato Y. Kawashima M. Furumichi M. Tanabe M. (2016). KEGG as a reference resource for gene and protein annotation. Nucleic Acids Res. 44 (D1), D457–D462. 10.1093/nar/gkv1070 26476454 PMC4702792

[B16] Kanehisa M. Furumichi M. Sato Y. Matsuura Y. Ishiguro-Watanabe M. (2025). KEGG: biological systems database as a model of the real world. Nucleic Acids Res. 53 (D1), D672–D677. 10.1093/nar/gkae909 39417505 PMC11701520

[B17] Knox C. Law V. Jewison T. Liu P. Ly S. Frolkis A. (2011). DrugBank 3.0: a comprehensive resource for 'omics' research on drugs. Nucleic Acids Res. 39 (Database issue), D1035–D1041. 10.1093/nar/gkq1126 21059682 PMC3013709

[B18] Krupaa R. J. Sankari S. L. Masthan K. M. K. Rajesh E. (2015). Oral lichen planus: an overview. J. Pharm. Bioallied Sci. 7 (Suppl. 1), S158–S161. 10.4103/0975-7406.155873 26015696 PMC4439656

[B19] Kurago Z. B. (2016). Etiology and pathogenesis of oral lichen planus: an overview. Oral Surg. Oral Med. Oral Pathol. Oral Radiol. 122 (1), 72–80. 10.1016/j.oooo.2016.03.011 27260276

[B20] Kurt S. Gürkan Ç. G. Keleş Tezal G. Ç. Çiftçi A. Gürgör P. N. Güler Ş. (2019). Histopathological and biochemical evaluation of the effect of paeoniflorin on the periodontium during and after periodontitis formation in rats. Arch. Oral Biol. 102, 135–140. 10.1016/j.archoralbio.2019.04.006 31005686

[B22] Lodi G. Manfredi M. Mercadante V. Murphy R. Carrozzo M. (2020). Interventions for treating oral lichen planus: corticosteroid therapies. Cochrane Database Syst. Rev. 2 (2), CD001168. 10.1002/14651858.CD001168.pub3 32108333 PMC7047223

[B23] Louisy A. Humbert E. Samimi M. (2024). Oral lichen planus: an update on diagnosis and management. Am. J. Clin. Dermatol 25 (1), 35–53. 10.1007/s40257-023-00814-3 37713153

[B24] Lu R. Zhang J. Sun W. Du G. Zhou G. (2015). Inflammation-related cytokines in oral lichen planus: an overview. J. Oral Pathol. Med. 44 (1), 1–14. 10.1111/jop.12142 24329772

[B25] Lu C. Wu C. Ghoreishi D. Chen W. Wang L. Damm W. (2021). OPLS4: improving force field accuracy on challenging regimes of chemical space. J. Chem. Theory Comput. 17 (7), 4291–4300. 10.1021/acs.jctc.1c00302 34096718

[B26] Marabi M. H. Yari K. Mozaffari H. R. Hatami M. (2024). Assessment of TNF-Alpha (-857 C/T) gene polymorphism in oral Lichen planus disease: a case-control study. Health Sci. Rep. 7 (4), e2014. 10.1002/hsr2.2014 38572118 PMC10988235

[B28] Mutafchieva M. Z. Draganova-Filipova M. N. Zagorchev P. I. Tomov G. T. (2018). Oral Lichen planus - known and unknown: a review. Folia Med. Plovdiv. 60 (4), 528–535. 10.2478/folmed-2018-0017 31188760

[B29] Ni J. Yang D. Song L. Li C. (2016). Protective effects of paeoniflorin on alveolar bone resorption and soft-tissue breakdown in experimental periodontitis. J. Periodontal Res. 51 (2), 257–264. 10.1111/jre.12305 26179445

[B30] Rosas Jimenez J. G. Fabian B. Hummer G. (2024). Faster sampling in molecular dynamics simulations with TIP3P-F water. J. Chem. Theory Comput. 20 (24), 11068–11081. 10.1021/acs.jctc.4c00990 39668361 PMC11672673

[B31] Rosignoli S. di Paola L. Paiardini A. (2023). PyPCN: protein contact networks in PyMOL. Bioinformatics 39 (11), 1367–4811. 10.1093/bioinformatics/btad675 37941462 PMC10641099

[B32] Ru J. Li P. Wang J. Zhou W. Li B. Huang C. (2014). TCMSP: a database of systems pharmacology for drug discovery from herbal medicines. J. Cheminform 6, 13. 10.1186/1758-2946-6-13 24735618 PMC4001360

[B33] Selaru C. A. Parlatescu I. Milanesi E. Dobre M. Tovaru S. (2023). Impact of altered lipid profile in oral Lichen planus. Maedica (Bucur) 18 (1), 12–18. 10.26574/maedica.2023.18.1.19 37266475 PMC10231171

[B34] Spirito F. Dioguardi M. Caponio V. C. Ambrosino M. Lo Muzio E. Lo Muzio L. (2024). Oral Lichen planus in children: a systematic review. Med. Oral Patol. Oral Cir. Bucal 29 (2), e152–e162. 10.4317/medoral.25938 38288854 PMC10945876

[B35] Stelzer G. Plaschkes I. Oz-Levi D. Alkelai A. Olender T. Zimmerman S. (2016). VarElect: the phenotype-based variation prioritizer of the GeneCards suite. BMC Genomics 17 (Suppl. 2), 444. 10.1186/s12864-016-2722-2 27357693 PMC4928145

[B36] Szklarczyk D. Nastou K. Koutrouli M. Kirsch R. Mehryary F. Hachilif R. (2025). The STRING database in 2025: protein networks with directionality of regulation. Nucleic Acids Res. 53 (D1), D730–D737. 10.1093/nar/gkae1113 39558183 PMC11701646

[B37] Takeuchi A. Koga K. Tokita Y. Matsumoto T. Satake E. Taguchi A. (2020). The effects of tokishakuyakusan, a traditional Japanese medicine (Kampo), ferulic acid and paeoniflorin, on human endometriotic stromal cells and peritoneal macrophages. J. Reprod. Immunol. 139, 103104. 10.1016/j.jri.2020.103104 32172005

[B39] Wang X. Wang C. Ji B. Wang J. Zheng M. Song L. (2026). Multimodal pre-training models of molecular representation for drug discovery. Natl. Sci. Rev. 13 (1), nwaf495. 10.1093/nsr/nwaf495 41536313 PMC12798728

[B40] Weaver C. T. Elson C. O. Fouser L. A. Kolls J. K. (2013). The Th17 pathway and inflammatory diseases of the intestines, lungs, and skin. Annu. Rev. Pathol. 8, 477–512. 10.1146/annurev-pathol-011110-130318 23157335 PMC3965671

[B41] Wei M. Wang L. Su X. Zhao B. You Z. (2026). Multi-hop graph structural modeling for cancer-related circRNA-miRNA interaction prediction. Pattern Recognit. 170, 112078. 10.1016/j.patcog.2025.112078

[B42] White C. M. Chamberlin K. Eisenberg E. (2019). Curcumin, a turmeric extract, for oral lichen planus: a systematic review. Oral Dis. 25 (3), 720–725. 10.1111/odi.13034 30614166

[B43] Xu X. Liu Y. Feng L. Yang Y. S. Liu S. G. Guo W. (2021). Interleukin-6 released by oral Lichen planus myofibroblasts promotes angiogenesis. Exp. Ther. Med. 21 (4), 291. 10.3892/etm.2021.9722 33717234 PMC7885057

[B44] Yu F. Xu N. Zhou Y. Li B. Li M. Wang Q. (2019). Anti-inflammatory effect of paeoniflorin combined with baicalin in oral inflammatory diseases. Oral Dis. 25 (8), 1945–1953. 10.1111/odi.13171 31393636

[B45] Zhang L. Wei W. (2020). Anti-inflammatory and immunoregulatory effects of paeoniflorin and total glucosides of paeony. Pharmacol. Ther. 207, 107452. 10.1016/j.pharmthera.2019.107452 31836457

[B46] Zhang Z. Zhang Y. Zhao Z. Li P. Chen D. Wang W. (2022a). Paeoniflorin drives the immunomodulatory effects of mesenchymal stem cells by regulating Th1/Th2 cytokines in oral Lichen planus. Sci. Rep. 12 (1), 18678. 10.1038/s41598-022-23158-0 36333421 PMC9636377

[B47] Zhang Y. Luo M. Wu P. Wu S. Lee T. Y. Bai C. (2022b). Application of computational biology and artificial intelligence in drug design. Int. J. Mol. Sci. 23 (21), 1422. 10.3390/ijms232113568 36362355 PMC9658956

[B48] Zhao L. Chang Q. Huang T. Huang C. (2018). Paeoniflorin inhibits IL-1beta-induced expression of inflammatory mediators in human osteoarthritic chondrocyte. Mol. Med. Rep. 17 (2), 3306–3311. 10.3892/mmr.2017.8222 29257281

[B49] Zhou Y. Zhou B. Pache L. Chang M. Khodabakhshi A. H. Tanaseichuk O. (2019). Metascape provides a biologist-oriented resource for the analysis of systems-level datasets. Nat. Commun. 10 (1), 1523. 10.1038/s41467-019-09234-6 30944313 PMC6447622

[B50] Zhu S. Liu B. Q. Hao M. J. Fan Y. X. Qian C. Teng P. (2017). Paeoniflorin suppressed high glucose-induced retinal microglia MMP-9 expression and inflammatory response *via* inhibition of TLR4/NF-kappaB pathway through upregulation of SOCS3 in diabetic retinopathy. Inflammation 40 (5), 1475–1486. 10.1007/s10753-017-0571-z 28639050

