# Supplementary material for: Elucidating the molecular mechanisms of paeoniflorin intervention in oral lichen planus: a computational biology and bioinformatics–based research strategy
Source: Front Bioinform. 2026 Jul 17;6:1817487. doi: 10.3389/fbinf.2026.1817487 (PMC13423862; doi:10.3389/fbinf.2026.1817487)
Supplement: Supplementary file 2 [file Supplementaryfile1.pdf]

Elucidating the Molecular Mechanisms of Paeoniflorin Intervention in Oral Lichen Planus: A Computational Biology and Bioinformatics–Based Research Strategy

Tian Zhao[a,b,c,1], Qi Chen[a,b,c,1], Liang Yu[d,*]

[a] Stomatological Hospital, General Hospital of Ningxia Medical University, Yinchuan, Ningxia, 750004, China

[b] College of Stomatology, Ningxia Medical University, Yinchuan, Ningxia, 750004, China

[c] Ningxia Province Key Laboratory of Oral Diseases Research, Yinchuan, Ningxia, 750004, China

[d] General Hospital of Ningxia Medical University, Yinchuan, Ningxia, 750004, China

[1] The authors made the same contribution to this work and share the role of the first author.

[*]Correspondence authors: E-mail addresses:

w15378935368@163.com

Analysis of molecular docking results

Molecular docking of paeoniflorin against the eight core targets yielded the AutoDock Vina binding free energies summarized in Table 3. Overall, paeoniflorin adopts a multi-modal recognition paradigm that combines conventional and carbon hydrogen bonds, π-stacking, and hydrophobic contacts to achieve stable accommodation across diverse binding pockets. The key interaction profiles for each target are described below and compared with those of the corresponding reference inhibitors.

AKT1 (1UNQ). Paeoniflorin forms conventional hydrogen bonds with THR87, GLY16, and GLU85, complemented by carbon hydrogen bonds with LYS20, GLU17, ARG86, and THR87, and a Pi–alkyl contact with VAL83 (−7.91 kcal/mol). This extensive polar network contrasts with the binding mode of Ipatasertib (−7.31 kcal/mol), which establishes only one conventional hydrogen bond (GLU85) and relies on carbon hydrogen bonds and a Pi–alkyl interaction with ILE84. The richer hydrogen-bonding pattern of paeoniflorin may account for its higher predicted affinity for AKT1.

IL6 (1IL6). In the IL6 complex, paeoniflorin engages in carbon hydrogen bonds with SER37, ARG40, and GLU51, a Pi–Pi T-shaped contact with LEU33, and alkyl interactions with LYS171 and LEU33 (−7.30 kcal/mol). The reference inhibitor LMT-28 (−6.58 kcal/mol) forms a conventional hydrogen bond with GLU175, carbon hydrogen bonds with ASP34, ARG30, and ARG40, and alkyl contacts. The additional π-stacking provided by paeoniflorin likely contributes to its superior affinity.

MMP9 (1GKC). Paeoniflorin is anchored to MMP9 through conventional hydrogen bonds with ASP177, ARG162, and GLN126, together with carbon hydrogen bonds to

ASP201 and ILE198 and a Pi–Pi T-shaped stacking with GLY197 (−6.29 kcal/mol). Batimastat (−8.38 kcal/mol) displays a more extensive interaction network, including conventional hydrogen bonds with ILE198, HIS203, and GLN169, carbon hydrogen bonds with ASP177, Pi–Pi stacking with GLY197, and alkyl contacts with VAL167 and ARG162. Although paeoniflorin shows a lower binding energy, its multi-point polar contacts still indicate a meaningful fit within the MMP9 active-site cleft.

STAT3 (6NJS). Paeoniflorin makes conventional and carbon hydrogen bonds with HIS332 and MET331 (−9.27 kcal/mol). Stattic (−9.16 kcal/mol) forms a conventional hydrogen bond with THR515, carbon hydrogen bonds with ILE569, ASP570, HIS332, and MET331, and a Pi–cation interaction with ASP334 and ARG335. The comparable affinities are consistent with a conserved anchorage in the STAT3 recognition site, mediated by key polar residues.

TNF (1TNF). Paeoniflorin establishes conventional hydrogen bonds with SER147, ARG32, and ASN34, a carbon hydrogen bond with GLY148, and alkyl/Pi–alkyl contacts with ALA33, VAL91, VAL17, and ALA35 (−9.80 kcal/mol). SPD304 (−10.43 kcal/mol) engages SER147 and ARG32 via conventional hydrogen bonds and forms alkyl/Pi–alkyl interactions with HIS15, LEU93, VAL17, ALA35, and other residues. The slightly lower affinity of paeoniflorin is offset by a broader distribution of polar contacts along the hydrophobic face of TNF.

IL1B (1I1B). Paeoniflorin forms conventional hydrogen bonds with LYS103 and MET148, a carbon hydrogen bond with MET44, Pi–Pi T-shaped stacking with PHE42 and PHE112, and alkyl/Pi–alkyl interactions with LEU10, LEU6, and LEU69 (−8.96

kcal/mol). In comparison, MCC950 (−6.04 kcal/mol) only provides carbon hydrogen bonds (PHE46, LEU6, ASN108, LYS103), Pi–Pi T-shaped stacking (PHE42, PHE112), and an alkyl contact with LEU110. The additional conventional hydrogen bonds formed by paeoniflorin appear to be responsible for its substantially higher affinity.

PDE4B (1ZKL). Paeoniflorin is coordinated via conventional hydrogen bonds with HIS256, THR321, and HIS212, carbon hydrogen bonds with ASN260 and ASP362, a Pi–Pi T-shaped interaction with VAL259, and Pi–alkyl contacts with PHE384, LEU420, PHE416, and ILE323 (−7.22 kcal/mol). Rolipram (−6.19 kcal/mol) forms conventional hydrogen bonds with ASP253, HIS216, and ASP362, a Pi–Pi T-shaped contact with PHE416, and Pi–alkyl interactions with VAL380 and ILE323. The additional anchoring residues engaged by paeoniflorin are in line with its improved binding energy.

PTGS2 (5F19). Paeoniflorin forms conventional hydrogen bonds with HIS388 and LEU390, carbon hydrogen bonds with HIS386, TYR385, and GLN203, and alkyl/Pi–alkyl contacts with VAL202, LEU391, and VAL295 (−7.91 kcal/mol). Celecoxib (−10.26 kcal/mol) exhibits a conventional hydrogen bond with HIS388 and more extensive alkyl/Pi–alkyl networks involving VAL444, LEU391, and VAL295. The lower affinity of paeoniflorin relative to celecoxib is consistent with the latter's larger hydrophobic enclosure, yet the multi-point polar contacts of paeoniflorin still indicate a favorable fit within the PTGS2 cavity.

Taken together, these binding poses reveal a recurrent stabilization signature for paeoniflorin: conventional and carbon hydrogen bonds provide accurate anchoring, π–π or π–alkyl interactions confer site selectivity, and alkyl/van der Waals contacts ensure

hydrophobic complementarity. This convergent multi-target engagement, supported by quantitative affinity comparisons (Table 3), provides a structurally coherent basis for the proposed systems-level action of paeoniflorin against oral lichen planus and sets the stage for subsequent molecular dynamics validation

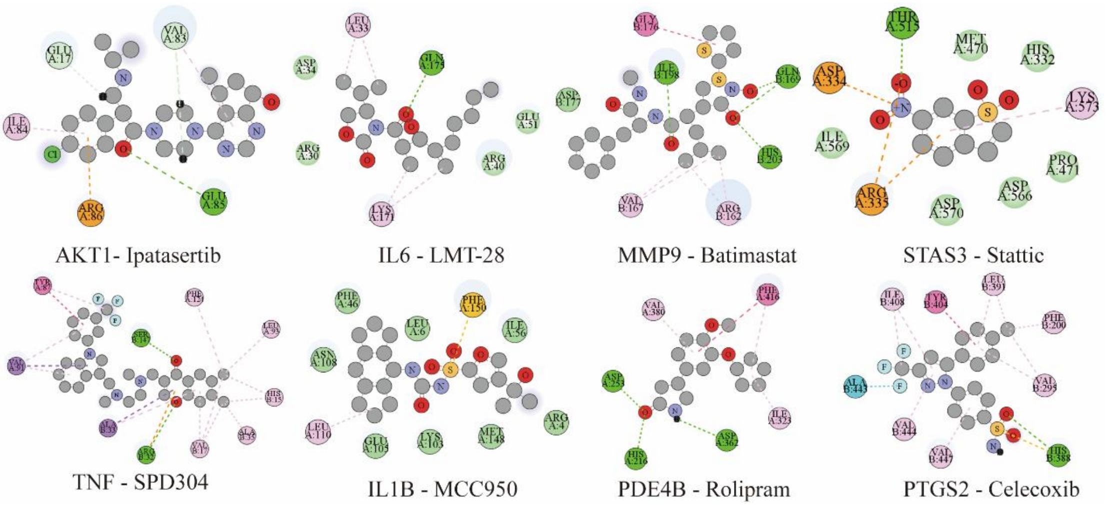

Figure S3: Refer to the molecular docking pattern diagram of the inhibitor and its corresponding target.

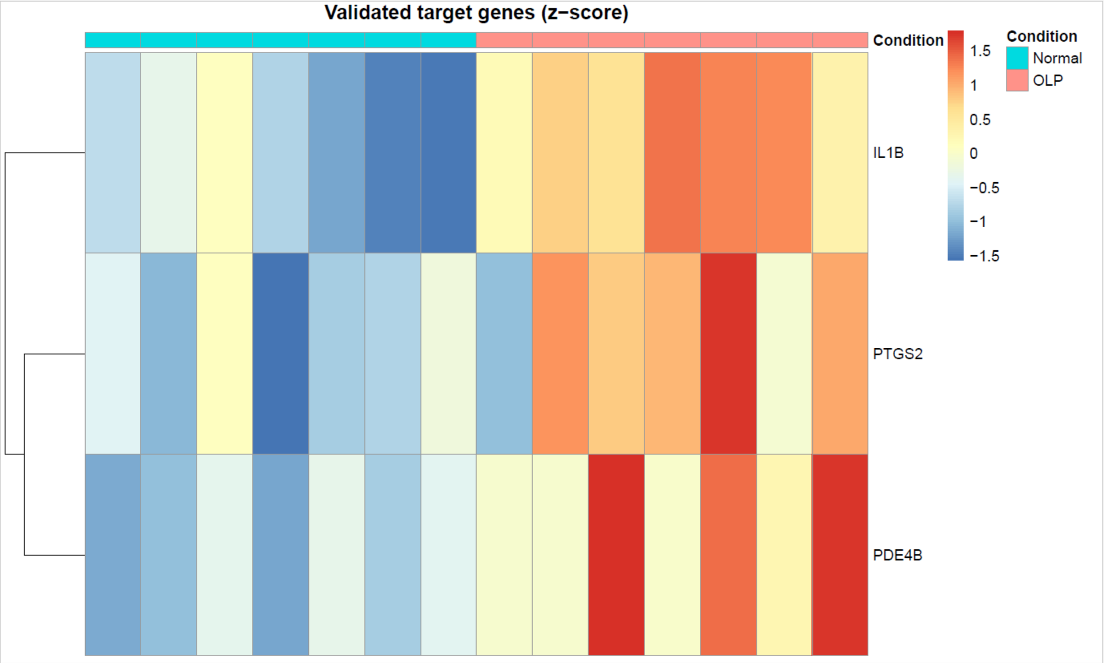

Figure S1: Transcriptomics differential gene analysis heatmap

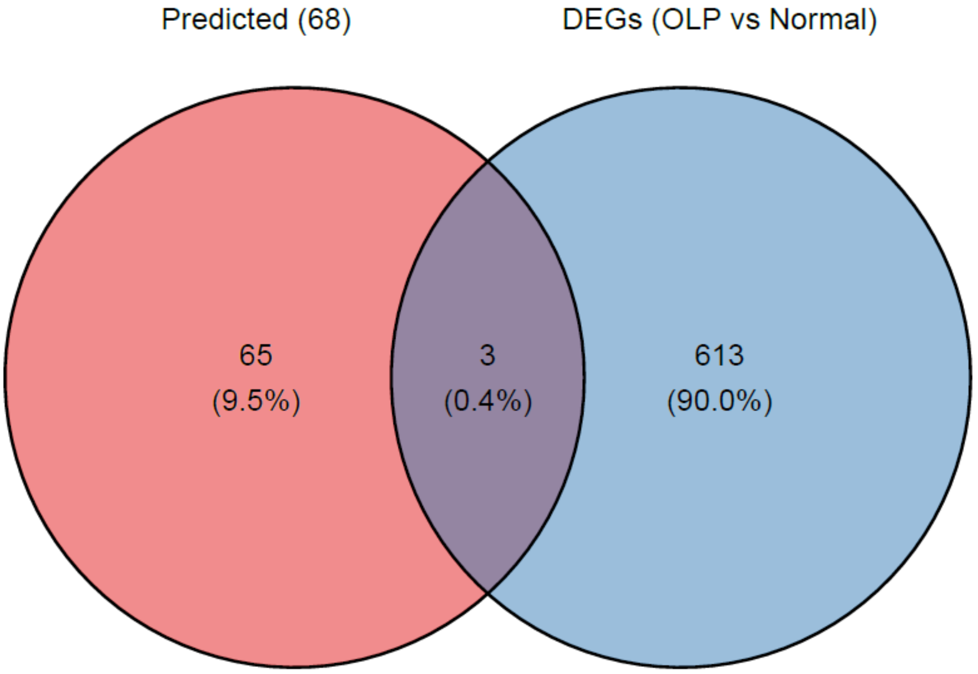

Figure S2: Venn diagram of differentially expressed genes in transcriptomics and network pharmacology-related targets

Table S1. Inter-trajectory variability of RMSD from three independent MD simulations

System	Component	Mean RMSD (nm)	Mean SD (nm)	Median SD (nm)
IL1B-1I1B	Paeoniflorin	0.1086	0.02900	0.02721
	Protein	0.2044	0.05167	0.04752
	Complex	0.3262	0.02088	0.01915
PDE4B-1ZKL	Paeoniflorin	0.1630	0.05307	0.05334
	Protein	0.1559	0.02133	0.02118
	Complex	0.2563	0.01838	0.01844
PTGS2-5F19	Paeoniflorin	0.09911	0.01824	0.01702
	Protein	0.21849	0.04158	0.04108

System	Component	Mean RMSD (nm)	Mean SD (nm)	Median SD (nm)
	Complex	0.31794	0.02681	0.02596
AKT1-1UNQ	Paeoniflorin	0.1557	0.03002	0.02621
	Protein	0.2446	0.02677	0.02425
	Complex	0.3766	0.02367	0.02015
IL6-1ALU	Paeoniflorin	0.2310	0.01886	0.01484
	Protein	0.2221	0.01912	0.01599
	Complex	0.3275	0.01471	0.01316
MMP9-1GKC	Paeoniflorin	0.1907	0.03602	0.03585
	Protein	0.2043	0.03637	0.03562
	Complex	0.2903	0.01668	0.01524
STAS3-6NJS	Paeoniflorin	0.1083	0.02170	0.01657
	Protein	0.2620	0.02438	0.02067
	Complex	0.3513	0.02022	0.01749
TNF-1TNF	Paeoniflorin	0.1293	0.03062	0.02587
	Protein	0.2305	0.02576	0.02419
	Complex	0.3377	0.01891	0.01836
